# Non-contrast myocardial perfusion assessment in porcine acute myocardial infarction using arterial spin labeled CMR

**DOI:** 10.1186/1532-429X-18-S1-O7

**Published:** 2016-01-27

**Authors:** Hung P Do, Venkat Ramanan, Terrence R Jao, Graham A Wright, Krishna S Nayak, Nilesh R Ghugre

**Affiliations:** 1 grid.42505.360000000121566853Department of Physics and Astronomy, University of Southern California, Los Angeles, CA USA; 2 grid.17063.33Physical Sciences Platform, Sunnybrook Research Institute, Toronto, ON Canada; 3 grid.42505.360000000121566853Department of Biomedical Engineering, University of Southern California, Los Angeles, CA USA; 4 grid.17063.33Department of Medical Biophysics, University of Toronto, Toronto, ON Canada; 5 grid.42505.360000000121566853Ming Hsieh Department of Electrical Engineering, University of Southern California, Los Angeles, CA USA

## Background

Following acute myocardial infarction (AMI), microvascular integrity and function may be compromised as a result of microvascular obstruction (MVO) and vasodilator dysfunction [1,2]. It has been observed that both infarct and remote myocardial territories may exhibit impaired myocardial blood flow (MBF) patterns associated with abnormal vasodilator response [3]. Arterial spin labeled (ASL) CMR is a novel non-contrast technique that can quantitatively measure MBF [4-6]. The aim of this study was to investigate the feasibility of ASL-CMR in assessing MBF in a porcine model of AMI.

## Methods

The Research Institute Animal Care Committee approved the protocol. The study involved a porcine model of AMI in which animals (N = 11) were subjected to a 90 min LAD occlusion followed by reperfusion. Animals underwent a CMR examination on a 3T scanner (MR750, GE Healthcare) at baseline (7 scans), at 1 day post-AMI (6 scans), and at 1 week post-AMI (5 scans). ASL-CMR, first-pass perfusion, and LGE imaging were all performed. First-pass perfusion and LGE were performed using product sequences while ASL-CMR was performed using our investigational pulse sequence [6] that uses flow-sensitive alternating inversion recovery (FAIR) labeling scheme and steady state free precession (SSFP) image acquisition with imaging parameters: TE/TR = 1.5/3.2 ms, FA = 50^0^, slice thickness = 10 mm, FOV = 18-24 cm, matrix size = 96 × 96, parallel imaging factor of 2. Labeling and imaging were triggered at mid-diastole. ASL-CMR was analyzed in a manner previously described to obtain global and per-segment MBF and physiological noise (PN); values were reported as mean ± SD. Segments with temporal signal-to-noise ratio (tSNR = MBF/PN) < 2 were excluded in the regional analysis.

## Results

**Figure 1:**
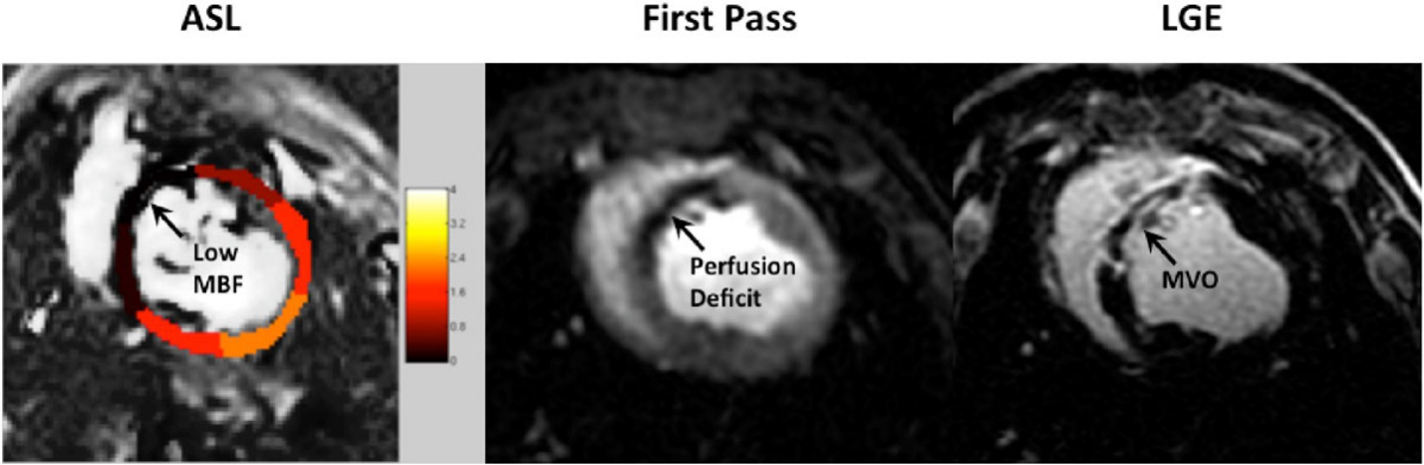
**ASL-CMR, first-pass CMR and LGE CMR from a representative animal at 1-week post-AMI**. In this example, MBF measurements were -0.07 ± 0.31 and 1.98 ± 0.20 m-bloodl/ml-tissue/min in the infarcted and remote regions, respectively. Near zero MBF is consistent with the resting perfusion defect on first-pass CMR and hypo-enhancement on LGE CMR (black arrows).

**Figure 2:**
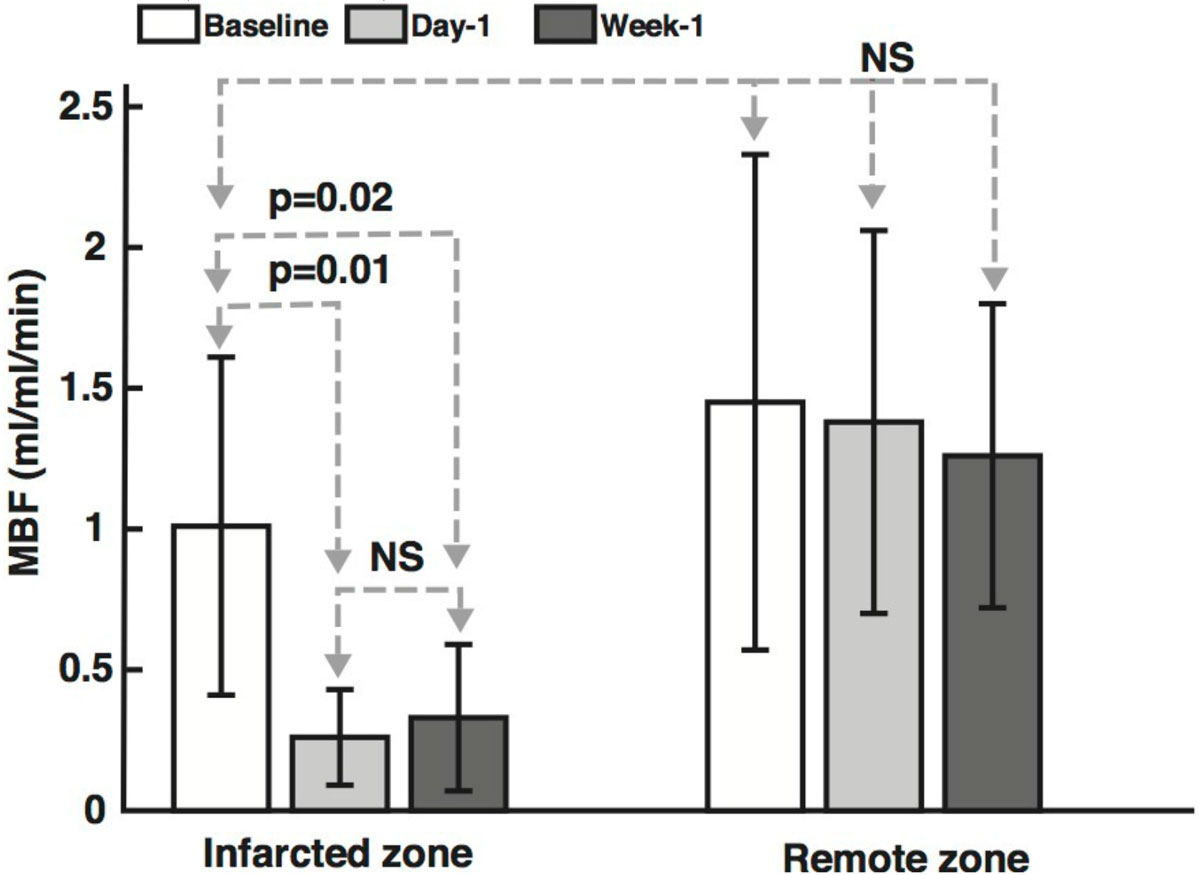
**MBF measured by ASL-CMR in infarcted and remote myocardial segments of all animals**. Error bars represent one standard deviation of physiological noise. MBF measurements from the post-AMI infarcted region were significantly lower (p < 0.05) than in healthy myocardium and remote myocardium at all time points. There was no significant difference between measured MBF in the remote region across time. NS: not significant.

Global and per-segment MBF in all baseline pigs was 1.12 ± 0.46 and 1.23 ± 0.76 (ml-blood/ml-tissue/min), respectively, consistent with literature values [7]. Global and per-segment PN in baseline animals was 0.13 ± 0.14 and 0.16 ± 0.09 (ml/ml/min), respectively, comparable to prior measurements in humans [6]. Consistent with the 90 min occlusion model [8], at day 1, all animals demonstrated a perfusion deficit on first-pass imaging and a MVO on LGE within the anterior infarcted territory. ASL measured low MBF values in the infarct zone, consistent with perfusion defect and MVO (see Figure 1). MBF measurements by ASL in the infarcted regions were significantly lower than in healthy myocardium (see Figure 2). There was no significant difference between measured MBF in the remote region across time. MBF measurements from the post-AMI infarcted region were significantly lower (p < 0.05) than in healthy myocardium and remote myocardium at all time points.

## Conclusions

Cardiac ASL is able to assess myocardial perfusion in a pig model of myocardial infarction. ASL is a potentially useful quantitative tool for longitudinally monitoring myocardial remodeling, particularly in the remote territory, which develops hypertrophy and fibrosis in the high-risk patients.

